# The influence of customer trust and artificial intelligence on customer engagement and loyalty – The case of the home-sharing industry

**DOI:** 10.3389/fpsyg.2022.912339

**Published:** 2022-08-04

**Authors:** Ying Chen, Catherine Prentice, Scott Weaven, Aaron Hisao

**Affiliations:** ^1^Department of Marketing, Griffith Business School, Griffith University, Brisbane, QLD, Australia; ^2^School of Business, University of Southern Queensland, Toowoomba, QLD, Australia; ^3^Staffordshire Business School, Staffordshire University, Stoke-on-Trent, United Kingdom; ^4^Griffith Institute for Tourism, Griffith Business School, Griffith University, Brisbane, QLD, Australia

**Keywords:** trust, AI, sharing economy, customer engagement, loyalty

## Abstract

Trust is an essential factor in online and offline transactions. However, the role of customer trust has received limited attention in the home-sharing economy. Drawing on the revised stimulus organism response model and trust transfer theory, this paper examines how customer trust in home-sharing hosts and platforms affects customer relationships, manifested in customer engagement and loyalty. As artificial intelligence (AI) is extensively utilized within home-sharing platforms to facilitate business operations and enhance the customer experience, this study also examines the influence of AI on customer trust and other related outcomes. The research was undertaken in China, with respondents who had used home-sharing platforms. Results from structural equation modeling show that customer trust had a significant positive relationship with customer engagement and loyalty. Customer engagement mediates the relationship between trust and loyalty, while AI may have a negative moderating effect between host trust and customer engagement and customer engagement and loyalty. The paper contributes to marketing, sharing economy and AI research. The work has implications for practitioners offering suggestions to develop marketing strategies for business growth and sustainability.

## Introduction

Trust is a critical issue for home-sharing businesses (Hossain, 2021). Unlike e-commerce, the sharing economy is not conducted in the virtual world. Contact within the real world (such as contact between hosts and guests, and Uber drivers and passengers) may damage goods or cause physical harm and potentially even the loss of life (Ter Huurne et al., 2017). Moreover, the regulatory uncertainty in this area increases the lack of security (Ranchordás, 2015). Trust helps overcome uncertainty, mitigate risk, and drive the success of C2C (customer-to-customer) platforms (McKnight and Chervany, 2001). Previous studies have proved that trust positively relates to behavior intention in Airbnb (Park and Tussyadiah, 2020). Furthermore, trust can bring other service outcomes (such as technical, functional and economic quality) (Doney et al., 2007; Watt and Wu, 2018). However, to the best of our knowledge, there is no research focused on trust outcomes in the home-sharing business.

Customer engagement and loyalty are highly related to service outcomes (economic quality). They have long been regarded as effective marketing strategies to maximize profitability and gain competitive advantages (Alvarez-Milán et al., 2018; Wang et al., 2021). Previous studies proved that trust could be a driver of customer engagement and loyalty (McKnight and Chervany, 2001; Kosiba et al., 2020), but whether this relationship still exists in the context of home-sharing platforms is still unknown. By extending the stimulus organism response model (SOR), we proposed that customer trust is the stimulus factor that can drive customer engagement (organism) and customer loyalty (response). Therefore, in this study, trust formation refers to how trust affects service outcomes (customer engagement and loyalty). Trust in the home-sharing industry is a hierarchical, two-fold construct, including trust in the platform and the host (Hawlitschek et al., 2016). We focused on trust in the hosts and trust in the platform and examined whether there is a trust transfer between them.

Artificial intelligence (AI) has been applied in home-sharing businesses and proved to be a cost-effective application (Ivanov and Webster, 2017). In home-sharing businesses, AI refers to smart devices or applications based on AI technologies, which are utilized to reduce costs and enhance the customer experience. For example, hosts use smart home devices (such as smart lock, smart thermostat, and home assistant) based on AI technology to reduce costs. AI can help foster trust through background checking and ID verification (Chen et al., 2021) and enhance customer engagement and loyalty by providing a memorable service experience (Prentice and Nguyen, 2020). Although AI is widely used in home-sharing businesses, the research in the sharing economy is in its infancy (Vlačić et al., 2021), and its contributions are underexplored. It is not clear what role AI plays in the home-sharing platform. Therefore, this study aims to respond to the following research questions: (1) whether trust in the host is related to the trust associated with home-sharing platforms, (2) how customer trust affects customer engagement and loyalty, (3) What role does AI plays in the home-sharing platforms.

This study adopts the revised stimulus organism response (S-O-R) theory and trust transfer theory to model the complex causal relationships. Research findings will contribute to marketing, sharing economy, and AI research. The study may contribute to marketing, sharing economy, and AI research. This study enriches the sharing economy literature by evaluating the role of AI and customer trust. It contributes to customer loyalty literature by bridging customer trust, engagement and loyalty in the sharing economy domain and using the extended SOR model as well as extending the AI literature by focusing on whether AI enhances customer relationships in the home-sharing platforms. The study also has important practical implications for home-sharing practitioners and other stakeholders. The following sections review the AI, trust, customer engagement, and customer loyalty literature and hypotheses development. Methodology, findings, discussion, and implications are then presented.

## Literature review

### The revised stimulus organism response model

**FIGURE 1:**
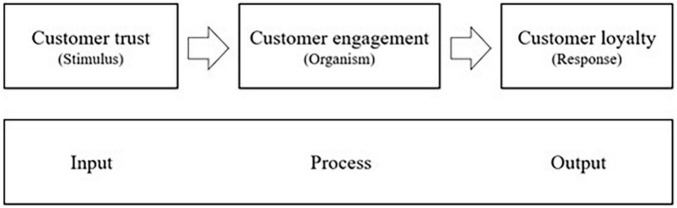
The extended S-O-R model.

The concept of the “Stimulus Organism Response” (SOR) model was developed from the theory of stimulus-response (Mehrabian and Russell, 1974). It indicates that environment and information signals play as stimuli and affect an individual’s responses, which affect behavior intentions (Bigne et al., 2020). The SOR model includes three components, which are stimulus (inputs), organism (processes), and response (outputs) (Mehrabian and Russell, 1974). It provides the theoretical foundation for consumer behavior studies (Kamboj et al., 2018; Islam et al., 2020; Amin et al., 2021). Scholars have extended the SOR model to fix their research context (Jang and Namkung, 2009; Kim et al., 2020). For example, Hossain et al. (2021) adopted this model and assumed that customers’ interactions would affect their flow experience and finally influence their trust, commitment and CRM performance. In this study, we extend the model to customer trust as the stimulus because it is an essential factor of the sharing economy. Customers can interact and reshape their evaluation. The organism is based on customers’ evaluations and perceptions and reflects the internal processes between the stimulus and customers’ final response (Islam and Rahman, 2017). Customer engagement is proposed as an organism. We suggest that customer engagement with the home-sharing platform will be affected by the trustworthiness of hosts and the platform. The SOR model’s response component is the outcome of customers’ actions and behavior, which is reflected as customer loyalty in this study. In association with the literature, the current study develops and tests an extended SOR model to predict customers’ behavior on home-sharing platforms (Shown in Figure 1). Besides, there are several studies focused on customer trust, engagement and loyalty from different perspectives. We summarized these key constructs in Table 1. Details of these components are introduced in the following sections.

**TABLE 1:** Summarize key constructs.

Construct	Definitions	Relationships
**Customer trust**	Trust refers to the personal bond between customers and the focal object (like a brand). Customers relied on the focal thing and believed that it acts in the customers’ best interest (Li et al., 2020; Ng et al., 2020). It is the confidence between exchange partners’ integrity and reliability. (Rather, 2018, 2020; Rather et al., 2018).	Driver: Trust is one of the drivers of customer engagement. Customer trust positively impacts loyalty (Bowden, 2009; Ng et al., 2020; Rather, 2020). Mediator: Customer trust fully mediated the influence of customer engagement on brand loyalty (Li et al., 2020).
**Customer engagement**	Customer engagement is defined from different perspectives: Interactive: Most definitions share the concept’s core interactive nature (Rather et al., 2022). It refers to individuals participating in and connecting with an organization’s offerings or activities (Rather et al., 2019). A behavior beyond transaction: Brodie et al. (2011) defined CE as “a psychological state, which occurs by interactive customer experiences with a focal object (e.g., a brand/destination)”. Van Doorn et al. (2010, p. 254) defined CE as “behaviors that go beyond transactions and may be specifically defined as a customer’s behavioral manifestations that have a brand or firm focus, beyond purchase, resulting from motivational drivers” (Rather, 2020).	Mediator role: Customer engagement mediates the relationship between place attachment and place authenticity on customer trust, loyalty, and cocreation (Rather et al., 2019). Influences on behavior intention: Customer engagement dimensions affect customer experience and cocreation, subsequently affecting revisit intent (Rather et al., 2022). The indirect effects of customer engagement dimensions on behavioral intentions *via* understanding and identification (Rather, 2020). Driven by many factors: Customer engagement is driven by satisfaction, positive emotions, and trust. It also increases satisfaction, commitment, trust, and loyalty (Rather, 2019; de Oliveira Santini et al., 2020).
**Customer loyalty**	Customer loyalty influences companies both in the long term and short term because it helps to gain new consumers and loyal customers likely to re-buying or re-patronizing products and services (Rather et al., 2018; Rather, 2019). Customer loyalty has two components: behavioral and attitudinal (Li et al., 2020).	Customer loyalty is a result of customer engagement. Customers who engage with a brand and service provider are expected to build positive attitudes instantly. Such perspectives draw on specific behavior such as loyalty or word of mouth (Rather et al., 2018; Ng et al., 2020)

### Trust in hosts and trust in the platform

Trust reflects that both parties show vulnerability to the other in uncertain circumstances and expect the other party to honor obligations (Ter Huurne et al., 2017). It is an essential element that sustains the development and the success of the sharing economy as it overcomes uncertainty and mitigates risk, ensuring long-term success (McKnight and Chervany, 2001; Botsman and Rogers, 2011). Scholars have proposed that the critical challenge in the sharing economy (e.g., home sharing, car sharing) is trust between strangers (Ter Huurne et al., 2017; Räisänen et al., 2020). The transaction model in the sharing economy requires interaction with unknown parties, which may damage goods, cause physical harm, or even cause loss of life. Regulatory uncertainty is a characteristic of the sharing economy (Ranchordás, 2015). Trust in home-sharing is a hierarchical, two-fold construct (Hawlitschek et al., 2016). According to Cheng (2016) three-level micro-meso-macro typology of the sharing economy, trust not only exists at the individual level (hosts and guests’ level) but also at the meso level (sharing platform level). Specifically, trust is not only a matter for the hosts but also relates to trust in the operating platform. Thus, trust in home-sharing platforms is interpreted as trust in the platform and the host. Trust in the platform suggests that customers believe the home-sharing platform protects against perceived risks. Trust in the host relates to the reliability and trustworthiness of the host.

There is a trust transfer between the trust for the underlying platform and the trust between stakeholders. The rationale for this proposition is based on Strub and Priest (1976) trust transfer theory, which suggests that trust can be transferred from different parties when the trustor has little or no experience (Strub and Priest, 1976). A trustworthy intermediary helps build the buyers’ trust by reducing potential risks within the e-commerce industry (Verhagen et al., 2006). Mittendorf (2017) has also demonstrated that this relationship exists in the Uber ride-sharing platform. Trust is transferred from the Uber platform to the drivers (Mittendorf, 2017). This discussion leads to the following hypothesis:

**H1:** Trust in the platform is positively and significantly related to trust in the host within the sharing economy.

### Trust and customer engagement

Customer engagement has been defined from different perspectives, for instance, a psychological process (Bowden, 2009), behavioral manifestation (Van Doorn et al., 2010) and a psychological state (Patterson et al., 2006). Patterson et al. (2006) defined customer engagement as a psychological state characterized by vigor, dedication, absorption, and interaction. We argue that customer engagement in the home-sharing platforms is a psychological state that reflects how customers’ psychological feelings about hosts and the sharing platforms. Customer engagement in the sharing economy reflects engagement with macroscopical economic actors (macro-level), engagement with platforms (Meso level), and engagement with service providers (macro-level) (e.g., Uber drivers and Airbnb hosts) (Breidbach and Brodie, 2017). This study focuses on the meso level and investigates engagement with home-sharing platforms.

Trust is a driver of customer engagement and is essential for long-term relationships because people prefer interactions in a trust relationship (Nahapiet and Ghoshal, 1998; Kosiba et al., 2020). Researchers pointed out that customers who trust online hotel websites are more likely to book online and commit to their relationship (Agag and El-Masry, 2016). A lack of trust is considered one of the leading causes of consumer non-engagement (Pavlou, 2003). Therefore, the following two hypotheses were proposed:

**H2a**: Trust in the host is positively and significantly related to customer engagement in the sharing economy.

**H2b**: Trust in the platform is positively and significantly related to customer engagement in the sharing economy.

### Trust and customer loyalty

Customer loyalty is a crucial factor and an indicator of organizational competitiveness and business success (Krumay and Brandtweiner, 2010; Best, 2013). It is critical for the home-sharing industry as it relies upon the return of both hosts and guests (Calixte et al., 2016). Moreover, customer loyalty reduces marketing costs as advertisements are less needed to attract repeat customers (Griffin and Herres, 2002). Customer loyalty includes attitudinal and behavioral loyalty. Attitudinal loyalty refers to the emotional attachment to an organization, while behavioral loyalty relates to the direct monetary benefit to the organization (Bandyopadhyay and Martell, 2007; Islam et al., 2020).

Trust can result in positive attitudes toward a brand (Jarvenpaa et al., 1999; Swan et al., 1999) and is a fundamental mechanism for building customer loyalty (Lee et al., 2015). Trust drives customer loyalty and the long-term success of C2C platforms (McKnight and Chervany, 2001). Trust also influences consumers’ purchase intention directly and indirectly (Grazioli and Jarvenpaa, 2000; Abubakar, 2016). In building trust, customers perceive positive outcomes and pursue for a long time positive results in the future, which reflects customer loyalty (Yap et al., 2012). When customers trust hosts and platforms in home-sharing platforms, they prefer to repurchase in the future. Two hypotheses were developed below:

**H3a:** Trust in the host is positively and significantly related to customer loyalty in the sharing economy.

**H3b:** Trust in the platform is positively and significantly related to customer loyalty in the sharing economy.

Research has shown that customer engagement impacts loyalty (Hollebeek, 2011; Prentice et al., 2018). Customer engagement is a psychological process that drives loyalty (Hapsari, 2017). Suppose customer engagement with a sharing platform is positive. In that case, it can lead to positive satisfaction, trust, and commitment and may result in further interaction and customer loyalty (Breidbach and Brodie, 2017). Thus, the following hypothesis was proposed:

**H4:** Customer engagement is positively related to customer loyalty in the home-sharing industry.

Many studies have also conceptualized and tested customer engagement as a mediator (Prentice and Nguyen, 2020). Engaged customers have a strong psychological connection with the brand or organization. This connection creates loyalty at and beyond purchase (Brodie et al., 2013; Abou-Shouk and Soliman, 2021). Loyalty is an attitudinal antecedent of customer engagement behaviors such as blogging, online shopping, and commenting. Factors that affect customer engagement also indirectly affect customer loyalty, indicating a mediated relationship (Van Doorn et al., 2010; Prentice and Nguyen, 2020). Therefore, the following hypotheses were proposed:

**H5a:** Customer engagement has a significant mediating effect between trust in the platform and customer loyalty.

**H5b:** Customer engagement has a significant mediating effect between trust in the host and customer loyalty.

### The role of artificial intelligence

Artificial intelligence (AI) has been gradually adopted by industry since it was first proposed in 1956 (Mellit and Kalogirou, 2008; Prentice et al., 2020a). It is the ability of a system to interpret and learn from external data and achieve specific goals and tasks (Libai et al., 2020). Prentice et al. (2020) defined AI as intelligent performance and behaviors by machines, computers, or robots to assist humans and businesses. With this in mind, and for the purpose of this review, we define it as applications or intelligent devices based on AI technologies (such as chatbots and voice/facial recognition systems), which are utilized in home-sharing platforms to reduce costs and enhance customer experience. Several home-sharing platforms have adopted various AI-based technologies, such as chatbots and facial recognition.

Ivanov and Webster (2017) suggest that AI adoption’s most significant financial benefit is labor cost savings. AI can reduce operating costs by 15% and increase revenue by 10% in the hospitality industry (ATM TEM, 2019). Research has established that 15 mins of work by an employee is equivalent to a minute of work by AI (Nam et al., 2020). Greater service availability results in greater ordering opportunities. Chatbots, for example, can provide a 24/7 service rather than the more limited 40-h weekly employee service. Chatbots can also serve multiple customers simultaneously, which is difficult to achieve with human-based services (Ivanov and Webster, 2017). AI also performs better in tedious, repetitive, and intellectually unchallenging tasks (Nam et al., 2020; Prentice et al., 2020a). Employee satisfaction can be improved by relieving them from boring and repetitive tasks. In home-sharing services, AI tools can simultaneously reduce repetitive tasks and help manage bookings and general inquiries. Listings that have adopted AI services have increased positive word of mouth as they may be perceived as high-tech accommodation (Doud, 2020).

AI has been adopted to enhance experiences for customers in the pre-transaction, transaction, to post-transaction stages (Lemon and Verhoef, 2016; Grover, 2019; Libai et al., 2020). By enhancing technology-enabled processes, AI reshapes the consumer journey and contributes to the customer relationship (Grover, 2019; Libai et al., 2020). AI can assist customers with purchases, travel choices, location preferences, and hotel payment options (Li et al., 2019; Prentice and Nguyen, 2021). AI experiences in home-sharing occur throughout the customer journey. In the pre-purchase stage, AI improves search rankings based on guest preferences (similar places that the guests click, location preferences) and provides immediate responses through conversational AI technologies (Predictive Analytics Team, 2020). During the purchase process, AI tools can facilitate the payment process. After check-in, customers can use AI tools to enhance security (smart doorbells in Xiaozhu) and enhance their entertainment experience (TV fruit in Tujia). Details of AI adopted in the home-sharing platform are shown in Table 2.

**TABLE 2:** Artificial intelligence adopted in the home-sharing industry.

Type	Examples	Platforms
Chatbots	Smartbnb	Airbnb
Voice recognition systems	Control for room temperature (Ecobee), Audio-visual interaction (TV fruit)	Airbnb, Tujia
Facial recognition systems	Guests check-in (“360” smart doorbell, Keycafe)	Xiaozhu, Airbnb
Analytics	Set best price (Beyond pricing, Price tips), guests’ background check (Trooly)	Airbnb

Artificial Intelligence-generated smart replies can increase trust between different stakeholders (Hohenstein and Jung, 2020). AI can foster trust through digital services such as background checking and ID verification (Chen et al., 2021). These services encourage customers to interact and engage with the sharing platform. With the help of AI, hosts can provide an outstanding service experience to customers, resulting in customer engagement. A good experience motivates customers to have more “physical, mental, social and emotional” engagement with the company (Carù and Cova, 2003; Prentice and Nguyen, 2020). Guests who experience outstanding services provided by AI tools tend to be more engaged with the platform. A good customer experience can lead to customer engagement and loyalty (Prentice et al., 2019). A memorable experience with AI can enhance the relationship between guests and the home sharing platform. These factors enhance the relationship between customer engagement and loyalty. This discussion leads to the following hypotheses:

**H6a:** AI significantly moderates the relationship between trust in the platform and customer engagement.

**H6b:** AI has a significant moderation effect on the relationship between customer engagement and customer loyalty.

**FIGURE 2:**
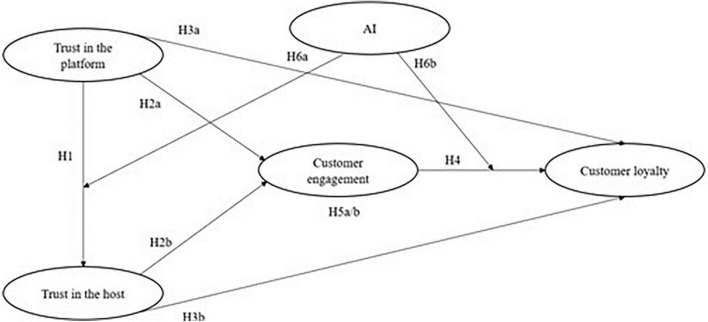
The research model.

The research framework is presented in Figure 2.

## Materials and methods

### Sample

Data were collected during May and June 2021. The sample for this study were Chinese residents over 18 years. Participants should have used a home-sharing platform within the last 2 years, and screening questions were asked to ensure these criteria. The data collecting time was during the pandemic; hence we could only conduct the online survey *via* social media due to restrictions on personal interactions. This paper employed virtual snowball sampling to access respondents. This method has been widely used as participants can share survey links within their social network (families, relatives, and friends) *via* social media platforms (e.g., WeChat, Weibo) (Baltar and Brunet, 2012). Virtual snowball sampling is respondent-driven; thus, it can generate a larger population with similar backgrounds (Heckathorn, 2011). This sampling is a good fit for this study as AI is an abstract concept that includes a variety of forms, although some forms may not be perceived as AI (Prentice et al., 2020). By using snowball sampling, participants can share the link with their relatives *via* social media platforms. At the same time, any relevant queries about AI can be answered directly by friends, which is more effective than responses from the researchers. There are also many home-sharing platforms in China, with many being local platforms only used in China (Cortese, 2020; State Information Center, 2020). The cited home-sharing platforms included Airbnb, Tujia (China’s largest home-sharing company), Xiaozhu, and other local Chinese platforms.

### Measurement

Measurement items were adapted from previous research. Factors relating to trust in the platform, trust in the host, customer engagement, customer loyalty, and AI were assessed using a 7-point Likert-type scale (with “1” indicating strongly disagree, and “7” strongly agree). Trust in the platform was measured using seven items (i.e., “I think hosts in this platform are reliable”) adapted from Mittendorf (2017) investigated different levels of trust in Uber as “trust in Uber” and “trust in drivers” to establish the relationship between trust and customer intentions. This measure was suitable as it was based on trust in Uber, which was a pioneer of the sharing economy (Mittendorf, 2017). The authors believe that trust in Uber and Airbnb may have similarities as both are leading share platforms and have a similar commercial model. Reliability was 0.88 on this scale. Similarly, trust in the host was measured with five items (i.e., “I trust the platform keeps my best interests in mind”) adapted from Jarvenpaa et al. (1999); Koufaris and Hampton-Sosa (2004), which was developed based on trust in an online company or store, and the scale reliability was 0.93.

Customer engagement was measured in three dimensions with 12 items, adapted from Cheung et al. (2011). This measure was suitable as it was developed for a social platform context and reflected customers’ vigor, absorption, and dedication to the platform. The reliability was 0.87, 0.90, and 0.90, respectively. Customer loyalty was adapted from Bobâlcă et al. (2012). The scale reliability was 0.92.

AI includes two dimensions, perceived usefulness (PU) and perceived ease of use (PEOU). The two dimensions have six items (i.e., “AI tool is easy to use.”) based on the work of Wixom and Todd (2005). As AI is technologically based, understanding AI uses in a home-sharing business does not focus on the technology itself but on how customers perceive it. The measure aims to reflect the degree of customer acceptance of the AI application. Reliability was 0.85 for this variable.

### Data collection procedure

The questionnaire was developed in English and translated into Chinese. The scales and items were then back-translated to English by a qualified translator to ensure validity (Sousa and Rojjanasrirat, 2011). 10 Ph.D students who had experience with home-sharing platforms participated in a pilot test to ensure response time and wording were appropriate. Issues with questions were modified based on the feedback. The study utilized Wenjuanxing^1^, similar to SurveyMonkey, and is China’s top market research tool (Kuo, 2018). The survey link was shared on social platforms such as WeChat, Weibo, and Facebook. Participants were guaranteed that the survey was anonymous and could stop the survey at any time. If all questions were answered, participants would receive a small token of appreciation (2 RMB). The questionnaire included two parts, with a total of 15 demographic questions being asked. The second section asked questions relating to trust, AI, customer engagement, and customer loyalty. A total of 546 responses were received after 1 month, of which 468 valid responses were selected after the exclusion of incomplete questionnaires.

Of the total retained sample, 57.1% were female, and 42.8% were male. Ages ranged from 18 to 56 and older. Nearly half of the respondents were of the 18−25 age group (48.3%), followed by the 26−35 age group (21.6%), the 36−45 age group (16.2%), and the 46−55 age group (9.4%). Only 4.5% of the respondents were 56 or more age group. Most of the respondents (59%) held a bachelor’s degree, and most participants were single (54.2%). Table 3 presents the demographic information.

**TABLE 3:** Profile of respondents (*N* = 468).

Characteristics		Percentage
Age	18−25	48.3
	26−35	21.6
	36−45	16.2
	46−55	9.4
	56 or more	4.5
Gender	Female	57.1
	Male	42.8
	Other	0.1
Education level	Bachelor’s degree	59
	High school	10.1
	Post-graduate	10.2
	Some college	20.7
Marital status	*Defacto* relationship	2.8
	Married	42.1
	Divorced	0.9
	Single	54.2

### Data analysis and results

#### Confirmatory factor analysis

Confirmatory factor analysis was used to assess the goodness-of-fit to assess the measurement model. AI and customer engagement were treated as second-order factors. The other three variables were treated as first-order factors. The results demonstrated a good model fit [χ^2^ = 1288.37, *df* = 545 (χ^2^/*df* = 2.36); CFI = 0.94; TLI = 0.93; RMSEA = 0.04]. Table 4 suggests that all items had a significant value of loading (greater than 0.50). The value of composite reliability (CR) and average variance extracted (AVE) was higher than 0.70 and 0.50, respectively, confirming adequate convergent validity of the measurement model (Fornell and Larcker, 1981). The correlations between the variables are presented in Table 5. The square root of the average variance extracted for each construct was greater than the correlation between the constructs, indicating discriminant validity.

**TABLE 4:** Confirmatory factor analysis results.

		Loading	Alpha	CR	AVE
Trust on platform			0.88	0.88	0.59
	Airbnb is trustworthy.	0.74			
	I trust Airbnb keeps my best interests in mind.	0.78			
	Airbnb will keep the promises it makes to me.	0.79			
	I believe in the information Airbnb provides me.	0.75			
	Airbnb wants to be known as one that keeps promises and commitments.	0.78			
Trust on hosts			0.93	0.93	0.65
	I trust the hosts using Airbnb.	0.80			
	I believe that the hosts of Airbnb are trustworthy.	0.82			
	I feel that the hosts of Airbnb are honest.	0.80			
	I feel that the hosts of Airbnb are reliable.	0.84			
	I feel safe while being served by hosts.	0.82			
	I don’t worry about crime issues about the hosts.	0.77			
	I don’t mind face-to-face contact with hosts during COVID-19.	0.79			
Customer loyalty			0.92	0.92	0.67
	I’m pleased to have used this platform.	0.82			
	It was a good idea to have stayed on this platform.	0.83			
	I will return to this platform.	0.85			
	I will say positive things about this platform.	0.84			
	I will recommend this platform to other people.	0.84			
	I will come back to this platform even if the price increases.	0.71			
AI			0.85	0.94	0.74
Ease of use			0.88	0.87	0.72
	AI tool is easy to use.	0.80			
	It is easy to get to do what I want it to do.	0.87			
	It is easy to operate.	0.87			
Usefulness			0.90	0.90	0.75
	Using AI tools improves my ability to make good decisions.	0.83			
	Using AI tools allows me to find home-sharing places more quickly.	0.89			
	Using AI tools enhances my effectiveness in booking rooms.	0.88			
Customer engagement			0.90	0.96	0.68
Vigor			0.87	0.87	0.63
	I can continue using this home-sharing platform for very long periods.	0.80			
	I feel strong and vigorous when I am using this home-sharing platform.	0.80			
	I devote much energy to this platform.	0.83			
	I try my hardest to perform well on this platform.	0.73			
Absorption			0.90	0.89	0.68
	Using this platform is so absorbing that I forgot about everything else.	0.82			
	Time flies when I am using this home-sharing platform.	0.80			
	I am rarely distracted when using this platform.	0.85			
	I am immersed in this platform.	0.82			
Dedication			0.90	0.90	0.75
	I found this platform full of meaning and purpose.	0.83			
	I am excited when using this home-sharing platform.	0.89			
	I am interested in this home-sharing platform.	0.87			

**TABLE 5:** Descriptive statistics, correlations, average variance extracted (AVE), and reliability.

	Trust in the platform	Trust in the host	Customer loyalty	Customer engagement	Artificial intelligence
Trust in the platform	**0.77**				
Trust in the host	0.65	**0.81**			
Customer loyalty	0.50	0.56	**0.82**		
Customer engagement	0.59	0.70	0.81	**0.82**	
Artificial intelligence	0.47	0.45	0.55	0.63	**0.86**

Bold values indicates the square root of the average variance.

#### Hypotheses testing

Structural equation modeling was used to test the hypotheses. The proposed model showed acceptable model fit: χ^2^ = 568.98; *df* = 183; (χ^2^/*df* = 3.11; CFI = 0.93; TLI = 0.93; RMSEA = 0.04). The value of R-square for customer engagement and customer loyalty were more than 38% and 54%, respectively, indicating a good fit for the model. H1 proposed that trust in the platform was positively related to trust in the host. The result shows that trust in the platform significantly affected trust in the host (β = 0.61, *p* < 0.001). H2a-b proposed that trust in the platform/trust in the host significantly affected customer engagement. The result shows that trust in a platform can significantly influence customer engagement (β = 0.21, *p* < 0.001) and loyalty (β = 0.47, *p* < 0.001), indicating both H2a and H2b were confirmed. The result shows that trust in the platform and trust in the host had a positive effect on customer loyalty (β = 0.11, *p* < 0.011; β = 0.14, *p* < 0.011). Thus, both H3a and H3b were supported. Further analysis proved H4 (β = 0.58, *p* < 0.001), which suggested that customer engagement positively affected customer loyalty. Results are shown in Table 6.

**TABLE 6:** Results of the proposed relationships.

Path			β	Sig	Hypotheses	Result
Trust in the platform	—>	Trust in the host	0.61	***	H1	Supported
Trust in the platform	—>	Customer engagement	0.21	***	H2a	Supported
Trust in the host	—>	Customer engagement	0.47	***	H2b	Supported
Trust in the platform	—>	Customer loyalty	0.11	**	H3a	Supported
Trust in the host	—>	Customer loyalty	0.14	**	H3b	Supported
Customer engagement	—>	Customer loyalty	0.58	***	H4	Supported
**R^2^**						
Trust on hosts			0.37			
Customer engagement			0.38			
Customer loyalty			0.54			
**Model fit**						
χ^2^ = 568.98, *df* = 183, χ^2^/*df* = 3.11, *p* < 0.001; CFI = 0.94; TLI = 0.93; RMSEA = 0.04						

***p < 0.001, **p < 0.01.

Hypotheses 5a and 5b proposed that customer engagement mediated the relationship between customer trust and loyalty. Gaskin and Lim’s (2018) plugin was used in AMOS to conduct a Sobel test. The results (see Table 7) show that the mediation effect of customer engagement was significant between trust in the platform and loyalty and between trust in the host and customer loyalty. As customer trust directly affects customer loyalty, the mediation of customer engagement was partially mediated for both trust in the platform and trust in the host. To confirm the mediation effect of customer engagement, the PROCESS macro 3.4 (Hayes, 2017) in SPSS 25.0 with 10, 000 bootstrapping samples was adopted. Results shown in Table 7 indicate that the 95% bootstrapping confidence intervals (BootLLCI) for the indirect effect on customer loyalty of trust in the platform was (CI = 0.20, 0.32), and trust in the host was (CI = 0.24, 0.35) not including 0. The result confirmed the mediation effect of customer engagement and supported H5a and H5b.

**TABLE 7:** Mediation test results.

Mediator	Between		Estimate	*P*-value	Lower	Upper
Customer engagement	Trust in the platform	Customer loyalty	0.12	0.001	0.07	0.18
Customer engagement	Trust in the host	Customer loyalty	0.27	0.001	0.21	0.34

**FIGURE 3:**
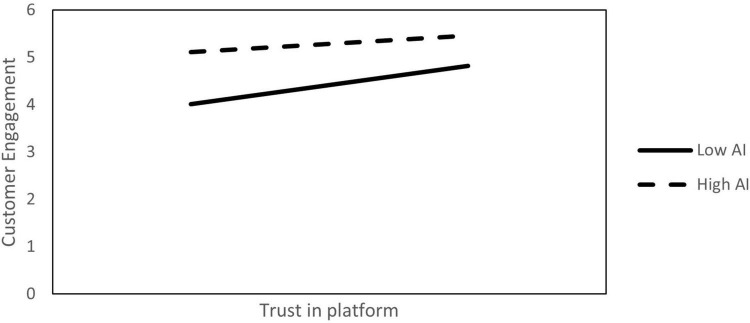
The moderation effect of AI on trust in the platform and customer engagement.

**FIGURE 4:**
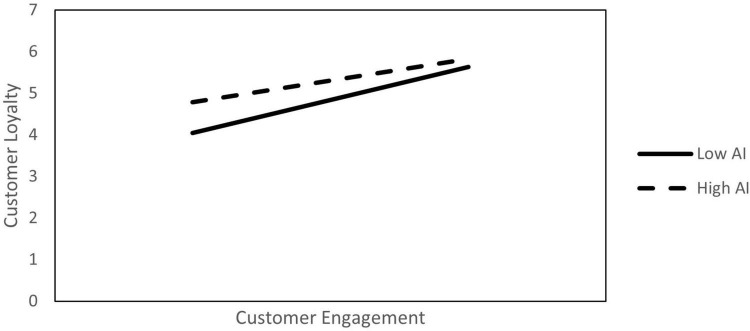
The moderation effect of AI on customer engagement and customer loyalty.

The moderating effect of AI was examined using AMOS. The results are presented in Table 8. The interaction effect between trust in the platform and AI on customer engagement was significant and supported H6a (β = −0.13, *p* < 0.001). Similarly, the moderation effect of AI on customer engagement and customer loyalty was also significant (β = −0.17, *p* < 0.001). The PROCESS macro with a bootstrapped sample of 10,000 (Hayes, 2017) was used again to confirm the moderating effect. The results show that AI significantly moderated the relationship between trust in the platform and customer engagement and customer engagement and customer loyalty (CI = −0.13, −0.04; CI = −0.17, −0.07; not including 0), supporting H6. Figures 3, 4 report the moderating effects of the three values of AI (mean level ± 1 standard deviation), providing simple slope plots of the interaction effect.

**TABLE 8:** Moderating test results.

Path			β	*P*
AI	—>	Customer engagement	0.43	***
AI	—>	Customer loyalty	0.20	***
Trust in the platform	—>	Customer engagement	0.28	***
Customer engagement	—>	Customer loyalty	0.55	***
Trust in the platform×_AI	—>	Customer engagement	−0.13	***
Customer engagement×_AI	—>	Customer loyalty	−0.17	***
R2				
Customer engagement			0.42	
Customer loyalty			0.57	

***p < 0.001.

## Discussion

Drawing on the SOR framework and trust transfer theory, this study investigated how customer trust in a platform and host could be transferred to customer engagement and loyalty and reflected on the role of AI during this process. It opted for home-sharing platforms in China as the research context and examined the mediating role of customer engagement and moderating role of AI. The results confirmed that customer trust could drive customer engagement and loyalty, while customer engagement played a mediating role in the proposed model. The moderating influence of AI exists, although its effects are negative. Details of findings are discussed in the following section.

### The influence of customer trust

This study found that trust in the platform positively influenced trust in the host, while customer trust was significantly related to customer engagement and loyalty. The significance of trust has been proved in the sharing economy, which includes the interaction of peers and multiple interactions in both online and offline settings (Luo and Zhang, 2016). When customers have trust in the accommodation sharing platform, hosts benefit from the transfer of trust as trustworthy transaction partners. This result is consistent with that of Mittendorf (2017); Park and Tussyadiah (2020). It confirms the trust formation process between platform providers and hosts and also emphasizes the important role of platform trustworthiness in customer decision-making.

Customer trust affects engagement and loyalty in a positive way, as shown in this study. It confirms that trust can procure interaction between customers and platform and is transferred to customer engagement (Strub and Priest, 1976; Kosiba et al., 2020). For example, Airbnb hosts are required to accept terms and conditions when they register as a host. In addition, Airbnb has an Airbnb ID Verification, which is used for a background checks and to uncover criminal convictions. As a result, Airbnb removes and blocks dishonest hosts who have criminal records (IGMS, 2020). Through these measures, customers have increased trust that the listing they are booking will be safe and reliable, which increases their willingness to interact with the platform. In terms of the relationship between trust and loyalty, this study shows that customer trust drives loyalty, confirming studies of McKnight and Chervany (2001); Abubakar (2016).

However, the influence of trust in hosts on customer engagement and loyalty was greater than that of trust in platforms. By using the home-sharing platforms, guests want to seek low price accommodation and authentic experiences through interaction with the local community (Park and Tussyadiah, 2020). Hosts are the first person guests contact within the whole community, which indicates that the impression of hosts is likely to determine guests’ evaluation of the home-sharing service. When guests choose an accommodation to live in, they check hosts’ profiles, ratings, and customer reviews to judge whether the hosts are trustworthy or not. Customers want to stay safe and have a good experience; thus, choosing a trusted host is a high priority.

### The mediation of customer engagement

The link between trust, customer engagement and customer loyalty has been well established in the relevant literature. This study proposed a different research context for home-sharing platforms and included customer engagement as the intervening customer-related outcome. The mediating role of customer engagement was confirmed and supported by previous research (Prentice and Nguyen, 2020; Abou-Shouk and Soliman, 2021). The establishment of mediation reflects a customer’s mental journey from customer trust, intention to interact, and loyalty behavior. The partial mediation effect indicates that trust in the platform and trust in the host have direct and indirect effects on customer loyalty. This relationship is largely attributed to the determinant role of customer trust. When customers do not trust the hosts or the platform, they may not attend the home-sharing business and never think about engagement or loyalty.

### The moderating role of artificial intelligence

AI has a negative moderating effect on trust in a platform and customer engagement, and customer engagement and loyalty. This finding indicates that customers with a high trust rating in a platform find that using AI does not increase engagement with the platform. Also, customers with high customer engagement do not find that using AI increases their loyalty. Two possible explanations exist: (1) the intangible characteristics of AI and (2) a preference for human interaction.

AI in the home-sharing context is an intangible service, making it difficult to assess and perceive. Prentice and Nguyen (2021) argued that robots and AI services have tangible and intangible forms. Tangible forms include humanoid and non-humanoid robots, while the intangible are online automated services. However, most AI tools are intangible without physical form, making them harder to perceive. While larger hotel chains can invest in humanoid robot services, it would be uncommon for a home-sharing business to have this investment capacity. It might also be noted that intangible AI can be confusing and frustrating for consumers, potentially resulting in negative feedback.

Further, a debate exists as to whether AI services are better than human interaction. Although the implementation of AI brings cost and time efficiencies, human interaction is still an important factor in the performance of service (Breidbach and Brodie, 2017). Customers prefer contact with human beings rather than robots (Wirtz et al., 2018; Prentice et al., 2020b). Moreover, in the home-sharing context, customers are seeking not only a room but also an authentic local experience (Lee, 2021). Customers prefer an authentic experience with contributions from the host, which is a key characteristic of the home-sharing economy. Unlike robot services or delivery services that are widely used in hotels, most AI tools in the home-sharing platforms focus on system support that hides behind, making it hard for customers to “feel it”. Thus, it is hard for customers to give positive feedback to AI services in the home-sharing industry.

## Implications, limitations, and future directions

### Theoretical implications

The theoretical implications for this study are trifold. First, this study contributes to customer loyalty literature using the extended SOR model. Second, it expands the sharing economy research. Third, AI literature is enriched by this study. Details of explanations are as follows.

The role of customer engagement has gotten much attention in the marketing domain. Many scholars focused on the relationship between customer engagement and loyalty. However, few studies have explored how customers influence customer loyalty in the sharing economy domain. Therefore, this study advanced customer loyalty literature by combining customer trust as an antecedent and including customer engagement as a mediator. The result shows that the bridge between customer trust, engagement and loyalty does exist in the home-sharing industry. Moreover, the current study extends the use of the SOR model by using customer trust as the stimulus, customer engagement as the organism and customer loyalty as the response. To the best of our knowledge, it is the first paper that extended SOR in this way, which may extend the usage of SOR in future studies.

This research extends the sharing economy literature by evaluating the role of AI and customer trust. The sharing economy research has primarily focused on sustainability or the impact on traditional industries (Narasimhan et al., 2018). This study focused on the outcomes of customer trust in the home-sharing industry, addressing a research gap. It provides a fresh perspective on how customer trust and AI contribute to the performance of home-sharing platforms. Furthermore, whilst most customer loyalty research focuses on marketing promotions and loyalty programs, this study provides a new view of the contribution of customer trust and AI to the customer experience.

As most AI research has tended to focus on AI techniques, this study extends the discussion to the domain of the home-sharing business and its influence on the performance of home-sharing platforms. Results show that AI plays a negative moderating role in the proposed relationships, which is inconsistent with previous research that AI enhances the relationship between customer satisfaction and loyalty (Prentice et al., 2020b). Thus, the adoption of AI is complicated within home-sharing platforms, causing researchers to revise the enhancement effect of AI under the background of sharing economy.

### Practical implications

The findings above show how these constructs influence customer loyalty on the home-sharing platforms, which indicates some insights for marketers and hosts.

The findings above show how these constructs influence customer loyalty on the home-sharing platforms, which indicates some insights for marketers and hosts. Customer trust significantly influenced customer engagement and loyalty, resulting in profit and platform development. Improving trust within platforms would benefit marketers, and hosts could consider how to make customers trust them more. For example, hosts who provide real rather than overly embellished photos can increase trustworthiness. Besides, this study confirms that customer engagement drives loyalty and mediates the relationship between trust and loyalty. Enhancing customer engagement within home-sharing platforms may have significant benefits for marketers. Airbnb users could generate content within the Airbnb community, giving users a sense of control and belonging within the platform. This form of community within a sharing platform can enhance customer interaction and engagement.

Although AI did not demonstrate a positive moderation effect on the relationships, this study still has value for practitioners. For example, while customers felt AI service in home-sharing was intangible and hard to perceive, other studies have suggested that human-like robots receive positive customer feedback (Prentice et al., 2020b). Platforms can provide human-like robots to hosts who won the “Superhosts” badge and see whether the robots will influence customers’ experience. Furthermore, the COVID-19 pandemic has increased the interaction risk between hosts and guests. It provides an opportunity for AI technology-based services, but how to capitalize on this opportunity to improve AI services requires further attention from marketers and scholars.

### Limitations and future directions

Some limitations must be acknowledged in this study. Respondents were Chinese citizens meaning the results may lack cultural diversity. The home-sharing platforms were also mostly local Chinese platforms, which may also lack comparison with other international platforms. The dimensions selected to measure AI were restricted to two dimensions and may inhibit a holistic understanding. Future studies will enrich AI dimensions and compare the relationship within home-sharing platforms from diverse backgrounds. The items used were translated and adopted from western scholars, and they may not be appropriate for Chinese respondents. Future studies will focus on the cultural influence on technology acceptance. This study focused on the customers’ perspective; future research may wish to explore the hosts’ perspective to gain further insights. In terms of customer trust, there are different trust preferences among different people. Some people tend to trust strangers and unfamiliar platforms more easily than others. Future studies should consider different personalities. As AI is hard to perceive, questionnaires may be limited in their capacity to reflect customers’ beliefs and attitudes toward AI. Future studies may consider other data collection methods (such as interviews or focus groups) or other research methods (such as meta-analysis or big data) to provide more meaningful insights into the usage of AI in the sharing economy. Lastly, we conducted this study during the pandemic. Future studies should measure this research model in post-pandemic to compare the results.

## Data availability statement

The raw data supporting the conclusions of this article will be made available by the authors, without undue reservation.

## Ethics statement

Ethical review and approval was provided by Griffith University. The project name is “The influence of artificial intelligence on customer engagement and customer loyalty in the sharing economy” (GU Ref No: 2019/963). Written informed consent from the patients/ participants or patients/participants legal guardian/next of kin was not required to participate in this study in accordance with the national legislation and the institutional requirements.

## Author contributions

YC wrote the first draft, collected the data, and did the data analysis. CP conceptualized the topic, formulated the model, structured the manuscript, assisted data analysis, and quality assurance. SW conceptualized the topic and revised the manuscript. AH helped to conceptualize the topic and revise the manuscript. All authors contributed to the article and approved the submitted version.

## Footnotes

1
www.sojump.com
